# Differences in mortality in patients undergoing surgery for infective endocarditis according to age and valvular surgery

**DOI:** 10.1186/s12879-020-05422-8

**Published:** 2020-09-25

**Authors:** Lauge Østergaard, Morten Holdgaard Smerup, Kasper Iversen, Andreas Dalsgaard Jensen, Anders Dahl, Sandra Chamat-Hedemand, Niels Eske Bruun, Jawad Haider Butt, Henning Bundgaard, Christian Torp-Pedersen, Lars Køber, Emil Fosbøl

**Affiliations:** 1 The Heart Center, Rigshospitalet, Copenhagen, Denmark; 2 Department of Cardiology, Herlev/Gentofte Hospital, Copenhagen, Denmark; 3 Department of Cardiology, Roskilde Sygehus, Zealand University Hospital, Roskilde, Denmark; 4 Institute of Clinical Medicine, Copenhagen University, Copenhagen, Denmark; 5 Clinical Institute, Aalborg University, Aalborg, Denmark; 6 Department of Cardiology and Clinical Research, Nordsjaellands Hospital, Hillerød, Denmark; 7 Department of Cardiology, Aalborg University Hospital, Aalborg, Denmark

**Keywords:** Infective endocarditis, Cardiac surgery, Endocarditis

## Abstract

**Background:**

Infective endocarditis (IE) is associated with high mortality. Surgery may improve survival and reduce complications, but the balance between benefit and harm is difficult and may be closely related to age and type of surgical intervention. We aimed to examine how age and type of left-sided surgical intervention modified mortality in patients undergoing surgery for IE.

**Methods:**

By crosslinking nationwide Danish registries we identified patients with first-time IE undergoing surgical treatment 2000–2017. Patients were grouped by age < 60 years, 60–75 years, and ≥ 75 years. Multivariable adjusted Cox proportional hazard analysis was used to examine factors associated with 90-day mortality.

**Results:**

We included 1767 patients with IE undergoing surgery, 735 patients < 60 years (24.1% female), 766 patients 60–75 years (25.8% female), and 266 patients ≥75 years (36.1% female). The proportions of patients undergoing surgery were 35.3, 26.9, and 9.1% for patients < 60 years, 60–75 years, and > 75 years, respectively. Mortality at 90 days were 7.5, 13.9, and 22.3% (*p* < 0.001) for three age groups. In adjusted analyses, patients 60–75 years and patients ≥75 years were associated with a higher mortality, HR = 1.84 (95% CI: 1.48–2.29) and HR = 2.47 (95% CI: 1.88–3.24) as compared with patients < 60 years. Factors associated with 90-day mortality were: mitral valve surgery, a combination of mitral and aortic valve surgery as compared with isolated aortic valve surgery, age, diabetes, and prosthetic heart valve implantation prior to IE admission.

**Conclusions:**

In patients undergoing surgery for IE, mortality increased significantly with age and 1 in 5 died above age 75 years. Mitral valve surgery as well as multiple valve interventions augmented mortality further.

## Background

Infective endocarditis (IE) remains a disease with an in-hospital mortality around 20% [1–3] and several studies have suggested that the incidence of IE is increasing - especially among patients aged > 75 years [2, 4–7]. It has been reported that surgery is performed in 23–52% of patients with IE from population-based cohorts [5, 8–10], however surgery in elderly patients may be challenging due to the burden of comorbidities and high perioperative risk. Several studies have described temporal changes of the epidemiologic characteristics of patients with IE and patients with IE now constitute an older population with a higher degree of comorbidities than previously [3, 5, 11, 12]. Little epidemiologic data exist on the prognosis following heart valve surgery for IE by age groups [3, 5, 11–15].

In a meta-analysis, including 16 studies examining risk scores for the assessment of in-hospital mortality in patients undergoing surgical treatment for IE, age, among other factors, was found to be associated with an increased risk of in-hospital mortality [16]. Further, age is included as a predictor in several surgical risk scores used for IE [17–20] but little data are present on the prognosis for IE patients > 75 years of age. Surgery on the mitral valve versus the aortic valve necessitates more extensive cardiac surgery, however differences in mortality between aortic and mitral valve surgery are sparsely described in patients with IE. The aim of this study was to examine mortality in patients undergoing surgical treatment for IE by age groups and to investigate differences by type of left-sided surgical valve intervention. Further, we aimed to examine factors associated with 90-day mortality.

## Methods

### Data sources

In Denmark, every citizen is provided with a personal identifier making it possible to link nationwide health registries [21]. For this study we used the Danish National Patient Registry, the Danish Population Registry, and the Prescription Registry. In brief, the Danish National Patient Registry holds information on every hospitalization since 1977 based on the discharge summary and diagnosis codes provided solely by the treating physician. One primary diagnosis is given (mandatory), while several secondary diagnoses may be given. The International Classification of Diseases (ICD-10) has been used since 1994. From 1996 and onwards surgical procedures were added to the registry using the Nordic Medico-Statistical Committee classification of surgical procedures. The registry has been described in detail previously [22]. The Danish Population Registry holds information on date of birth, date of death, and sex. The Danish Prescription Registry holds information on every prescription filled by a Danish pharmacy from 1994. The registry has been described in detail previously [23]. Information on medication given in-hospital is not available from the registries used.

### Study population, follow-up and outcome

Patients with first-time IE undergoing cardiac surgery from 2000 to 2017 were included. The study population was grouped in 1) patients < 60 years of age at IE admission, 2) patients aged 60–75 years, and 3) patients aged ≥75 years. In a supplementary analysis, the population undergoing left-sided valvular intervention was grouped in 1) isolated aortic valve surgery, 2) isolated mitral valve surgery, and 3) a combination of aortic and mitral valve surgery. Further, in supplementary material, analysis of cardiac valve intervention was stratified by age groups (1: < 60 years, 2: 60–75 years, 3: ≥75 years).

IE was defined from the following ICD-10 codes: I33, I38, and I398. Patients were required to have a hospital stay of minimum 14 days unless they died during the first 14 days of admission [24]. We accounted for transfers between hospitals and departments using a 24 h criteria for discharge/admission overlap. The positive predictive value of the ICD-10 codes for IE in the Danish National Patient Registry using the abovementioned criteria is 90% [25]. Heart valve surgery was defined from the Danish National Patient Registry using the procedure codes defined in Supplementary Table 1. Subclassification of the surgical valve procedure was conducted as follows: mechanical aortic valve replacement, bioprosthetic aortic valve replacement, other aortic valve replacement (xenograft, homograft, and aortic valve replacements classified as “other”), surgical aortic valve intervention without valve replacement, mechanical mitral valve replacement, bioprosthetic mitral valve replacement, and surgical mitral valve intervention without valve replacement.

Patients were followed from the date of heart valve surgery until death, December 2017, or a maximum of 5 years of follow-up, whichever came first. The primary outcome was all-cause mortality 90 days after valve surgery. Further, we report in-hospital and five-years mortality.

### Covariates

Medical history was assessed as a diagnosis of myocardial infarction, heart failure, atrial fibrillation/flutter, mitral or aortic valve disease, cardiac implantable electronic device, prosthetic heart valve prior to IE, renal disease, dialysis, peripheral vascular disease, cerebrovascular disease, cancer, chronic obstructive pulmonary disorder, and liver disease at any time prior to IE admission from the Danish National Patient Registry. Renal disease included patients with prior nephritic and nephrotic syndrome, patients with renal failure, anuria or oliguria, hypertensive renal disease with and without heart failure. Cancer was defined as a malignant neoplasm from any organ. Age and sex were assessed from the Danish Population Registry and concomitant pharmacotherapy was assessed from the Danish Prescription Registry as a filled prescription in a period of 6 months prior to IE admission. Diabetes was defined from the prescription of glucose lowering medication as done previously [26].

### Statistics

Baseline characteristics of the study population were presented in counts and percentages for categorical variables and with the median and 25 and 75 percentiles for continuous variables. Comparison of baseline characteristics between groups (1: < 60 years, 2: 60–75 years, 3: ≥75 years) were conducted using the chi-square test for categorical variables and the Kruskal-Wallis test for continuous variables. In-hospital mortality was assessed as the number of patients surgically treated who died during IE admission divided by the total number of patients surgically treated for IE by age groups. The chi-square test was used for investigating statistically significant difference between groups of in-hospital mortality. Mortality at 90 days and 5 years for the three age groups were plotted using Kaplan-Meier estimates. Associated risk of in-hospital mortality between age groups and by type of valve intervention was assessed in a multivariable adjusted logistic regression and 90 days mortality was assessed using Cox proportional hazard analysis. The following covariates were included in the models: type of surgical intervention during IE admission (mitral vs aortic vs mitral and aortic valve surgery), sex, heart failure, atrial fibrillation, age group, acquired mitral valve disease before IE admission, acquired aortic valve disease before IE admission, inserted cardiac implantable electronic device, prosthetic heart valve prior to IE admission, dialysis prior to IE admission, chronic obstructive pulmonary disease, diabetes, and calendar year periods. The assumption of proportional hazard was examined and for the investigation of 5 years mortality in patients undergoing left-sided valvular intervention the proportional hazard assumption was violated. A landmark analysis was conducted splitting the follow-up time at 90 days of follow-up. Calendar year was at first hand included as a continuous variable; however the assumption of linearity was not met, and the variable was categorized accordingly. Linearity of age was confirmed and to illustrate the associated risk of mortality with increasing age, a restricted cubic spline was modeled from the Cox regression model. The median age was used as reference value.

We identified factors at baseline associated with 90 days mortality using Cox proportional hazard analysis. Results are presented with a hazard ratio. The level of statistical significance was *p* < 0.05. Data management and statistical analysis was conducted using SAS software 9.4 (SAS Institute, Inc., Cary, NC, USA) and the statistical software R version 3.5.0 [27].

## Results

### Description of the study population

We identified a total of 7845 patients admitted for first-time IE in the period from 2000 to 2017, of which 1767 patients (22.5%) underwent cardiac surgery during IE admission; 735 (41.6%), 766 (43.4%), and 266 (15.1%) patients were < 60 years of age, 60–75 years, and ≥ 75 years, respectively. The proportion of patients with IE undergoing surgery was 35.3, 26.9, and 9.1% for the three age groups, respectively. The age group ≥75 years undergoing surgical intervention had a higher burden of comorbidities compared with the two younger study groups, Table 1. Of the total study population (1767 patients), isolated aortic valve surgery was conducted in 917 patients (51.9%), isolated mitral valve surgery in 498 patients (28.2%), and a combination of mitral and aortic valve surgery in 255 patients (14.4%), right-sided valve surgery in 42 patients (2.4%), and a combination of left- and right-sided surgery in 55 patients (3.1%). For patients undergoing left-sided surgical intervention (*n* = 1670), baseline characteristics are shown in Supplementary Table 2 and overall, it was seen that patients undergoing mitral valve surgery more often were female.

**Table 1:** Baseline characteristics for patients undergoing valve surgery for IE by age groups

	All	< 60 years	60–75 years	≥75 years	
Number	1767	735	766	266	
Age, median (IQR)	63 [52.7, 71.6]	50 [41.4, 55.6]	67.7 [63.8, 71.3]	78.2 [76.3, 80.3]	< 0.001
Female, N (%)	471 (26.7)	177 (24.1)	198 (25.8)	96 (36.1)	0.0006
Medical history prior to IE admission, N (%)					
AMI	102 (5.8)	26 (3.5)	52 (6.8)	24 (9.0)	0.001
Heart failure	220 (12.5)	56 (7.6)	116 (15.1)	48 (18.0)	< 0.001
Afib	241 (13.6)	35 (4.8)	142 (18.5)	64 (24.1)	< 0.001
Mitral valve disease	165 (9.3)	52 (7.1)	86 (11.2)	27 (10.2)	0.02
Aortic valve disease	455 (25.7)	107 (14.6)	227 (29.6)	121 (45.5)	< 0.001
CIED	71 (4.0)	16 (2.2)	34 (4.4)	21 (7.9)	< 0.001
Prosthetic heart valve	228 (12.9)	51 (6.9)	104 (13.6)	73 (27.4)	< 0.001
Renal disease	123 (7.0)	57 (7.8)	53 (6.9)	13 (4.9)	0.29
Dialysis	28 (1.6)	12 (1.6)	14 (1.8)	< 4	0.48
Peripheral vascular disease	149 (8.4)	34 (4.6)	78 (10.2)	37 (13.9)	< 0.001
Cerebrovascular disease	158 (8.9)	38 (5.2)	80 (10.4)	40 (15.0)	< 0.001
Cancer	191 (10.8)	44 (6.0)	104 (13.6)	43 (16.2)	< 0.001
COPD	128 (7.2)	21 (2.9)	77 (10.1)	30 (11.3)	< 0.001
Liver disease	57 (3.2)	28 (3.8)	24 (3.1)	5 (1.9)	0.31
Diabetes	182 (10.3)	49 (6.7)	95 (13.1)	34 (12.8)	< 0.001
Medication six months prior to IE admission, N (%)					
Diuretics	543 (30.7)	120 (16.3)	301 (39.3)	122 (45.9)	< 0.001
Beta blockade	409 (23.1)	100 (13.6)	223 (29.1)	86 (32.3)	< 0.001
RAS inhibition	547 (31.0)	133 (18.1)	305 (39.8)	109 (41.0)	< 0.001
Lipid lowering medication	428 (24.2)	82 (11.2)	243 (31.7)	103 (38.7)	< 0.001
Corticosteroids	138 (7.8)	42 (5.7)	69 (9.5)	26 (9.8)	0.02
Aspirin	367 (20.8)	70 (9.5)	175 (24.1)	104 (39.1)	< 0.001
Anticoagulants	298 (16.9)	74 (10.1)	147 (20.2)	61 (22.9)	< 0.001
Antibiotics	899 (50.9)	359 (48.8)	374 (51.4)	149 (56.0)	0.13

*IE* infective endocarditis, *IQR* interquartile range, 25th and 75th percentile, *AMI* acute myocardial infarction, *Afib* atril fibrillation/flutter, *CIED* cardiac implantable electronic device, *COPD* chronic obstructive pulmonary disease, *RAS* renin-angiotensin system

The percentages of the qualitative variables are shown in parentheses

### Mortality according to age groups

**Fig. 1:**
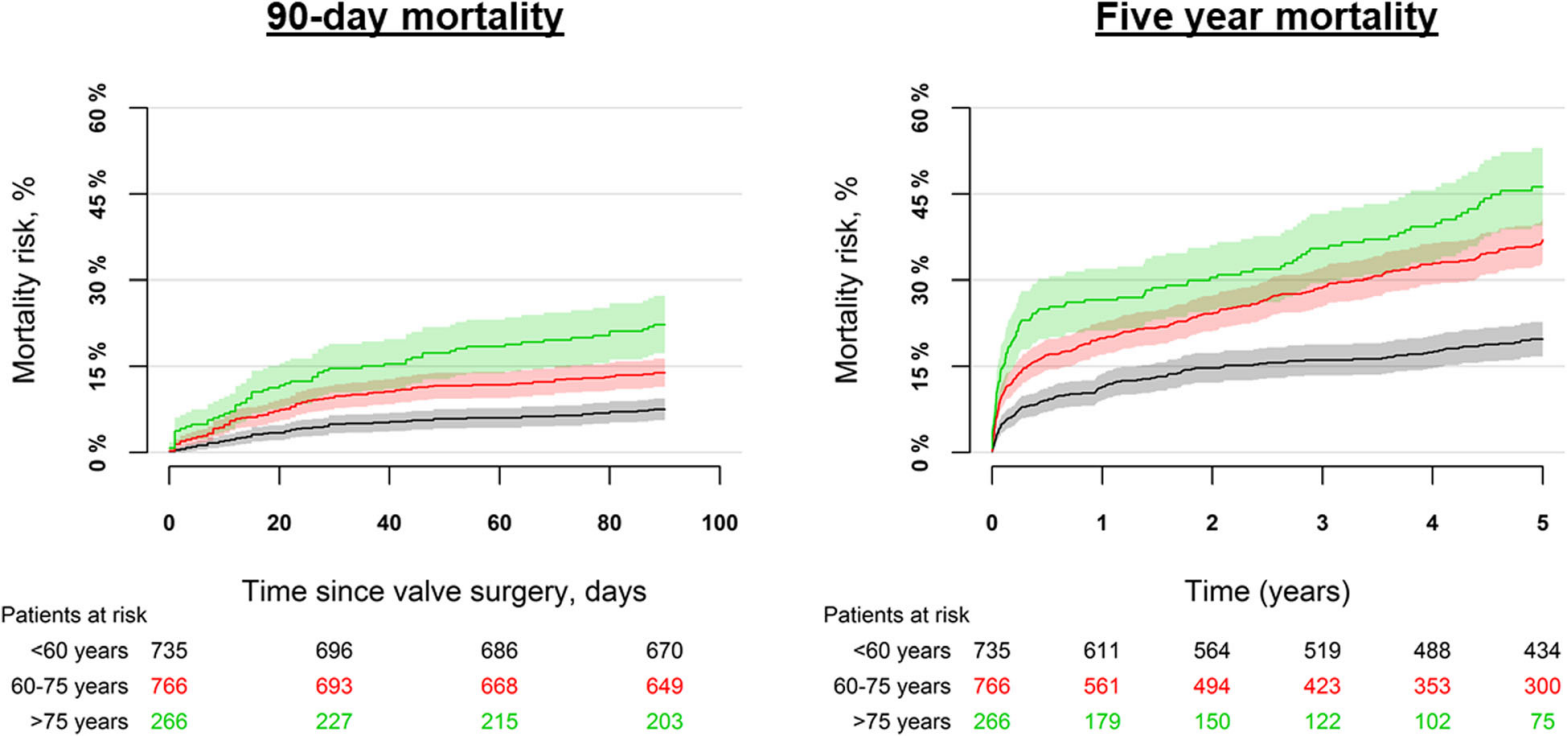
Mortality by age groups. The figure shows the 90-day mortality (left panel) and 5 year mortality (right panel) by age groups

The in-hospital mortality was overall 11.6% and for age groups it was 6.4%, 13.6%, and 20.3% for patients < 60 years, 60–75 years, and ≥ 75 years of age, respectively (*p* < 0.001), Supplementary Table 3. In adjusted analysis, the associated odds of in-hospital mortality were higher among patients 60–75 years and ≥ 75 years, OR = 2.00 (95% CI: 1.36–2.94) and OR = 3.33 (95% CI: 2.10–5.28), respectively as compared with patients < 60 years. The mortality at 90 days of follow-up was 7.5%, 13.9%, and 22.3% for patients < 60 years, 60–75 years, and ≥ 75 years of age (*p* < 0.001), respectively while the 5 years mortality was 19.7%, 37.0%, and 46.2% (*p* < 0.001), respectively, Fig. 1 and Supplementary Table 3. In adjusted analyses, patients 60–75 years and patients ≥75 years were associated with a higher mortality, HR = 1.84 (95% CI: 1.48–2.29) and HR = 2.47 (95% CI: 1.88–3.24), respectively as compared with patients < 60 years.

### Mortality according to type of left-sided valvular surgery

**Fig. 2:**
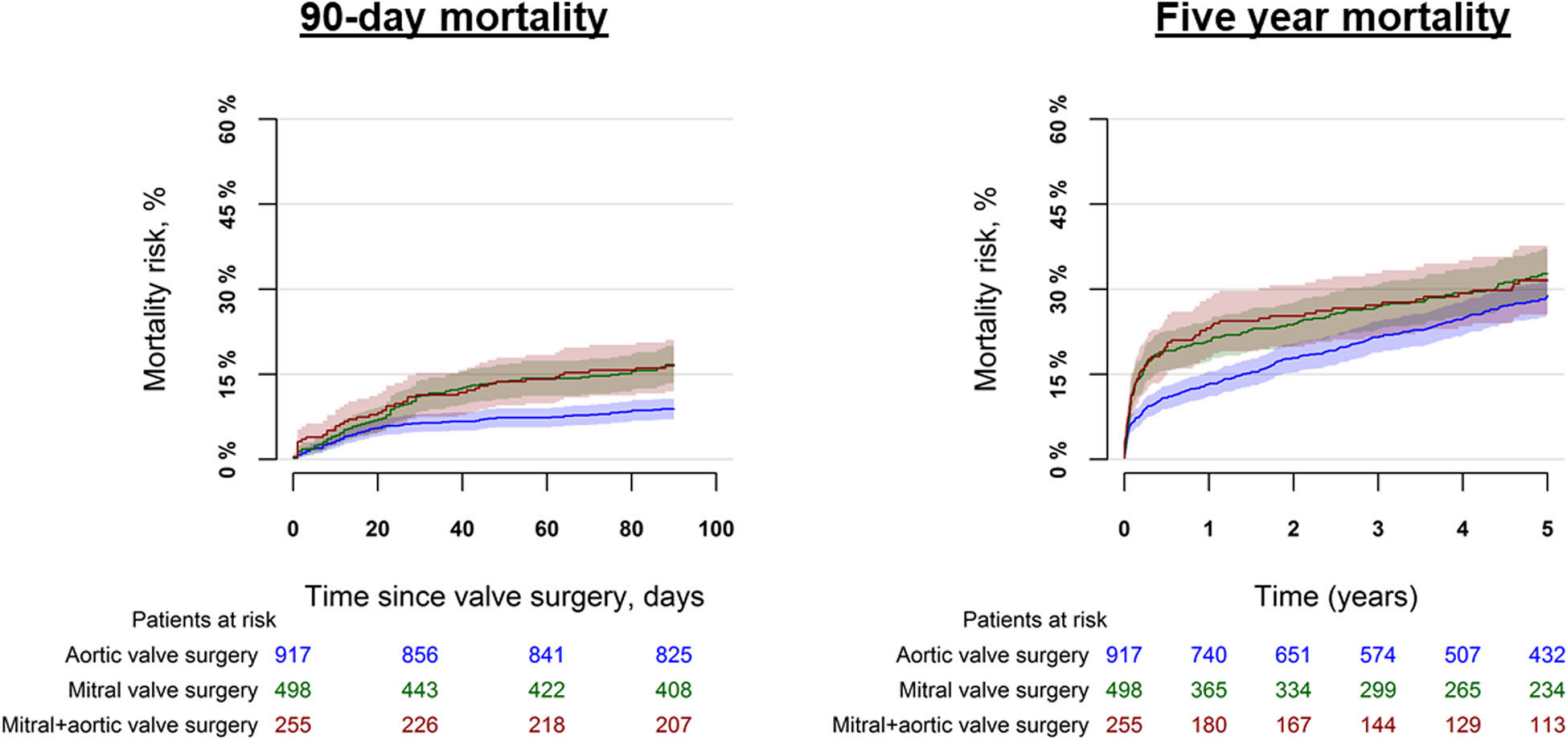
Mortality by type of left-sided valve surgery. The figure shows the 90-day mortality (left panel) and 5 year mortality (right panel) by type of left-sided valve intervention

For patients undergoing isolated aortic valve surgery, isolated mitral valve surgery, a combination of aortic and mitral valve surgery, the in-hospital mortality was 8.1%, 14.3%, and 18.0% (*p* < 0.001), respectively, Supplementary Table 3. In an adjusted analysis, the associated odds of in-hospital mortality were higher among patients undergoing isolated mitral valve surgery OR = 2.15 (95% CI: 1.42–3.26), a combination of aortic and mitral valve surgery OR = 2.99 (95% CI: 1.94–4.62) as compared with patients undergoing isolated aortic valve surgery. The 90 days mortality for these groups were 8.9%, 16.7%, and 16.5% (*p* < 0.001), while the 5 years mortality was 28.9%, 32.7%, and 31.6% (*p* = 0.07), respectively, Fig. 2 and Supplementary Table 3. In an adjusted analysis, we found differences in the associated risk of mortality by type of valve intervention with 0–90 days of follow-up, HR = 2.04 (95% CI: 1.43–2.92) and HR = 2.12 (95% CI: 1.43–3.12) for patients undergoing isolated mitral valve surgery, a combination of aortic and mitral valve surgery, respectively as compared with patients undergoing isolated aortic valve surgery. In a landmark analysis, with 90-days to 5 years of follow-up, the associated risk of mortality was: HR = 1.00 (95% CI: 0.72–1.38) and HR = 0.90 (95% CI: 0.61–1.34), respectively as compared with patients undergoing isolated aortic valve surgery.

### Mortality according to type of valvular surgery by age groups

Mortality of isolated aortic valve surgery, isolated mitral valve surgery and a combination of aortic and mitral valve intervention for the three age groups are presented in the Supplementary material. The overall results presented in the Supplementary material showed that for isolated aortic valve surgery, the 90 days mortality were 5.4%, 9.8%, and 15.6% for patients < 60 years, 60–75 years and ≥ 75 years, respectively, Supplementary Table 3. For isolated mitral valve surgery, the 90 days mortality were 10.8% for < 60 years, 18.5% for 60–75 years, and 29.6% ≥75 years. For a combination of aortic and mitral valve surgery the 90 days mortality were 9.0% for < 60 years, 16.0% for 60–75 years, and 36.3% for ≥75 years, Supplementary Table 3. Results of multivariable adjusted analyses are shown in Supplementary material, Supplementary Figures 2, 3, 4 and confirm unadjusted results showing an increased associated mortality with increasing age.

### Factors associated with 90-day mortality

**Fig. 3:**
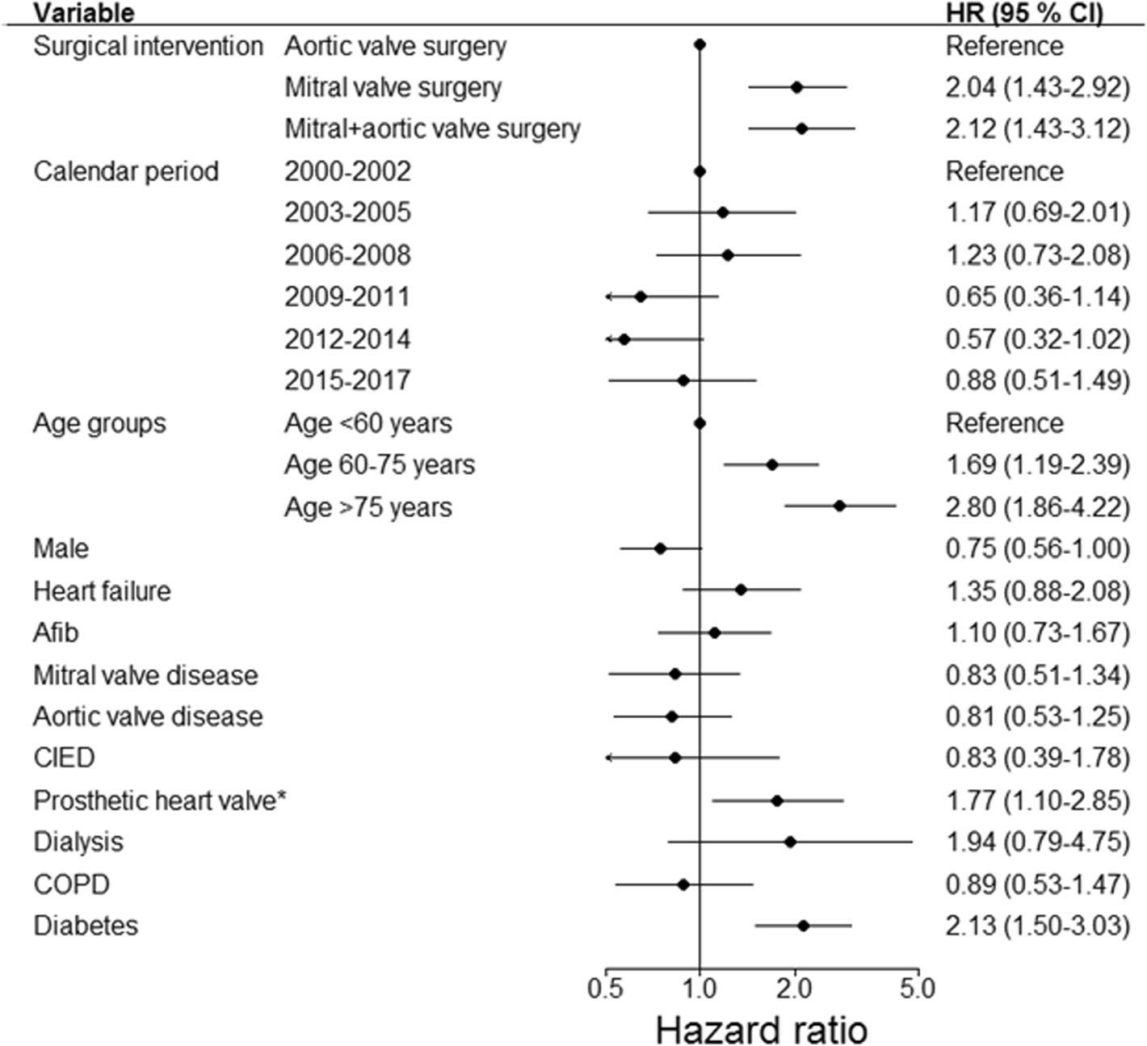
Associated risk of 90-day mortality after valve surgery for IE. The figure shows the associated risk of 90-day mortality. *Prosthetic heart valve prior to IE admission compared with patients with native valves. Afib: atrial fibrillation/flutter, CIED: cardiac implantable electronic device, COPD: chronic obstructive pulmonary disease

We found that isolated mitral valve surgery, and a combination of aortic and mitral valve surgery were factors associated with an increased risk of 90-day mortality as compared with patients undergoing isolated aortic valve surgery, Fig. 3. Further, increasing age, prosthetic heart valve implantation prior to IE admission, and diabetes were factors associated with an increased risk of 90-day mortality, Fig. 3. Including age as a continuous variable in the multivariable model was associated with an increased risk of mortality (per 1 year increase), HR = 1.04 (95% CI: 1.02–1.05). In Supplementary Figure 1, the associated risk of mortality is plotted according to increasing age.

## Discussion

This study investigated the associated in-hospital, 90-day, and 5 years mortality in patients undergoing valvular surgery for IE by age groups and type of left-sided valvular intervention. Our study had four major findings. First, the proportions of patients undergoing surgery were 35.3%, 26.9%, and 9.1% for patients < 60 years, 60–75 years, and > 75 years, respectively. Second, the 90-day mortality was around 1/15 patients, 1/7 patients, and 1/5 patients for < 60 years, 60–75 years, and ≥ 75 years of age, respectively. Third, in adjusted analyses, the mortality at 90 days was higher in patients undergoing mitral valve surgery, and a combination of mitral and aortic valve surgery as compared to isolated aortic valve surgery. Further, increasing age, prosthetic heart valve implantation prior to IE admission, and diabetes were factors associated with an increased 90-day mortality. Fourth, for patients ≥75 years undergoing a combination of aortic and mitral valve surgery, 36% died within 90 days of surgery.

### Proportion of patients with IE undergoing surgery

Our study, based on nationwide data, showed that 22.5% of patients with IE underwent cardiac valve surgery during IE admission. In comparison, a study from the International Collaboration on Endocarditis (ICE) identified that 57% of patients with left-sided IE underwent surgery [28]. Patients included in the ICE cohort were highly selected from tertiary centers and it has previously been shown that these patients more often undergo surgical intervention [29, 30]. Data from a large prospective cohort from 156 centers, 40 countries included 3116 cases of IE and found that 51.2% underwent cardiac surgery. Around 8.0% of the included cases were recruited from low-volume centers [8]. Further, from a French population-based study it was identified that 49% of 390 patients with IE underwent early valve surgery [11]. Our findings are based on data from an unselected patient cohort and provide novel results on a patient population sparsely described. A study from 14 French hospitals included 120 patients with IE ≥75 years and identified that 15.8% underwent valvular surgery [31]. A Spanish study from four tertiary centers identified that 17.6% (*N* = 6) of octogenarian patients underwent surgery.

### Mortality by age groups

Characteristics of patients with IE have changed over the last decades and patients with IE are older at the time of diagnosis, the majority is male, and more patients have a prosthetic heart valve [12, 32]. The decision of surgical intervention may be difficult in elderly patients and our results shed light on the prognosis subsequent to valve surgery in patients with IE. Our study showed that patients ≥75 years who underwent surgery, the in-hospital and 90-day mortality was tripled compared with patients < 60 years. Several risk-scores have been developed to predict in-hospital mortality in patients with IE with an indication for surgery and age constitutes an important parameter in these risk scores [17–20, 33]. However, no data from nationwide registries have explored the actual in-hospital, intermediate, and long-term mortality in patients undergoing surgery for IE across age groups. A prospective cohort of patients with IE from Spain, mainly from tertiary centers, identified 3120 patients with IE from 2008 to 2015. In patients ≥80 years the in-hospital mortality was 34.7% and age ≥ 80 years were associated with an increased mortality. From our data, an in-hospital mortality of 20.3% was identified among patients ≥75 years [34]. A single-center study from Australia, investigated 465 patients undergoing cardiac surgery for IE and found, among other factors, that increasing age, active bacterial endocarditis, and a high European System for Cardiac Operative Risk Evaluation score were associated with an increased risk of in-hospital mortality [35]. A previously mentioned Spanish study with data from tertiary centers concluded that age was not an independent predictor of in-hospital mortality [36]. This study population may be biased by selection since only patients from tertiary centers were included [36].

### Mortality by type of valvular intervention

Our findings showed that mitral valve surgery, and a combination of mitral and aortic valve surgery were not only associated with significantly higher in-hospital and 90-day mortality in general but also across age groups. Careful considerations of surgical indication in patients undergoing mitral valve surgery are necessary. In a landmark analysis, we identified that the increased associated risk of mortality for patients undergoing mitral valve surgery as compared with isolated aortic valve surgery was found during the initial 90 days of follow-up after valvular surgery. After the initial 90 days of follow-up, no differences in the associated risk of mortality was identified between by type of valvular surgery.

The authors studying the ICE cohort found that an increment in the Society of Thoracic Surgeons (STS) score of mortality risk was associated with an increased six-month mortality, however type of valvular surgical intervention was not assessed [28]. Our findings suggest that valve location is critical to assess the in-hospital and 90-day mortality of patients with IE. Mitral valve surgery is a major cardiac surgical intervention as compared with isolated aortic valve surgery and previous studies have found a higher mortality in patients undergoing mitral valve surgery as compared with aortic valve surgery [37, 38]. A study from the STS Adult Cardiac Surgery Database, identified that among 13,617 surgical procedures for IE, 40.2% was conducted on the mitral valve, 35.7% on the aortic valve, and 19.9% was conducted on more than one valve [33]. For the assessment of morbidity and mortality, the authors identified, among other factors, that age > 60 years and that surgery on more than one valve were factors associated with major morbidity and postoperative mortality [33]. Our study is in line with these findings and supplements current knowledge with unique data on mortality by age groups and type of valve intervention [33]. Several single-center studies from Sweden, Spain, Switzerland, and the United States, have identified an in-hospital mortality between 9 and 26% and a 5 years mortality between 22.4 and 25.0% in patients undergoing surgery for IE [39–42]. Further, a single-center study from Canada investigated patients with IE undergoing a combination of aortic and mitral valve surgery, including 90 patients with an all-cause mortality at 5 years of follow-up at 32% [43]. From nationwide data, we identified an all-cause mortality of 31.6% with up to 5 years of follow-up in patients undergoing a combination of aortic and mitral valve surgery. We found that 9.1% of all patients with IE ≥75 years underwent surgery and among the surgically treated patients undergoing a combination of aortic and mitral valve surgery more than one third died within 90 days of surgery (Supplementary material).

### Strengths and limitations

The main strength of this study is that data were provided from reliable nationwide registries ensuring an unselected, large cohort of patients undergoing surgery for IE with long-term follow-up. However, some limitations were present. First, we had no data from echocardiography which could have helped characterize the infectious spread on cardiac structures. Second, data on the indication of surgery and microbiological etiology was not accessible from the registries used. Third, risk factors for 90-day mortality were assessed in multivariable adjusted analyses, however residual confounding may exist, which may have influenced the estimates presented.

## Conclusion

In patients undergoing surgery for IE, we identified a 90-day mortality at 1/15 patients, 1/7 patients, and 1/5 patients for < 60 years, 60–75 years, and ≥ 75 years of age, respectively. Mitral valve surgery and combined mitral and aortic valve surgery were associated with an increased risk of 90-day mortality. Age, prosthetic heart valve implantation prior to IE admission, and diabetes were factors associated with an increased risk of 90-day mortality. For patients ≥75 years undergoing a combination of aortic and mitral valve surgery around 36% died within 90 days of surgery.

## Supplementary information

**Additional file 1: Supplementary Figure 1.** Associated risk of 90-day and 5 years mortality by increasing age. The figure shows the multivariable adjusted associated risk of 90-day and 5 years mortality by age. Reference value is the median age.

**Additional file 2: Supplementary Figure 2.** Mortality by age groups for isolated aortic valve surgery. The figure shows the 90-day mortality (left panel) and 5 year mortality (right panel) by age groups for surgical intervention on the aortic valve.

**Additional file 3: Supplementary Figure 3.** Mortality by age groups for isolated mitral valve surgery. The figure shows the 90-day mortality (left panel) and 5 year mortality (right panel) by age groups for surgical intervention on the mitral valve.

**Additional file 4: Supplementary Figure 4.** Mortality by age groups for a combination of aortic and mitral valve surgery. The figure shows the 90-day mortality (left panel) and 5 year mortality (right panel) by age groups for surgical intervention on a combination of the aortic and mitral valve.

**Additional file 5.** Supplementary material, Results continued.

**Additional file 6: Supplementary Table 1.** Codes.

**Additional file 7: Supplementary Table 2.** Baseline characteristics for patients undergoing left-sided valve surgery for IE.

**Additional file 8: Supplementary Table 3.** The table shows the mortality by sub groups.

## Data Availability

It is not allowed to publish data from Statistics Denmark due to rules of anonymity. Data used for this study was anonymized before use. The Danish National Patient Registry, the Danish Population Registry, The Danish Cause of Death Registry, and the Danish Prescription Registry were used [21–23, 44]. All registries are administered by Statistics Denmark. The databases are closed. The project was approved by the Danish Data Protection Agency with approval number: P-2019-401.
