# Supplementary figures and images for: Differences in mortality in patients undergoing surgery for infective endocarditis according to age and valvular surgery
Source: BMC Infect Dis. 2020 Sep 25;20:705. doi: 10.1186/s12879-020-05422-8 (PMC7519559; doi:10.1186/s12879-020-05422-8)

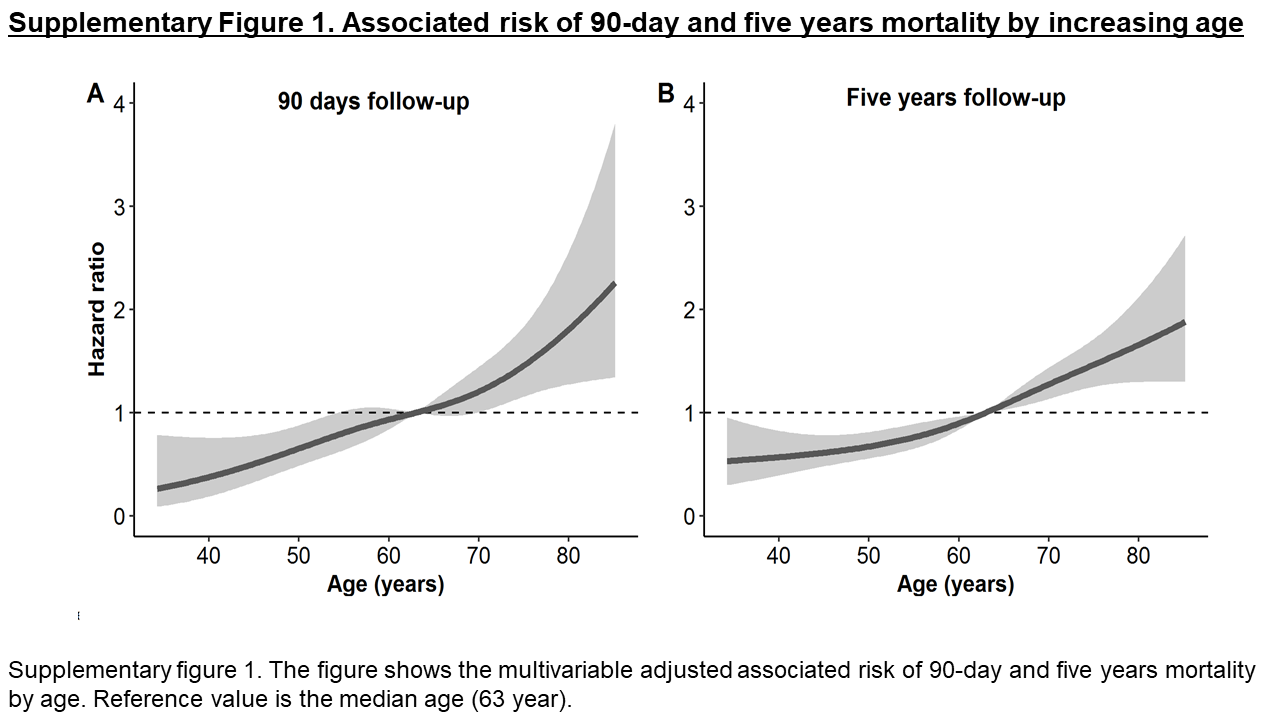

Supplement: Supplementary file 1 — Additional file 1: Supplementary Figure 1. Associated risk of 90-day and 5 years mortality by increasing age. The figure shows the multivariable adjusted associated risk of 90-day and 5 years mortality by age. Reference value is the median age. [file 12879_2020_5422_MOESM1_ESM.tif]

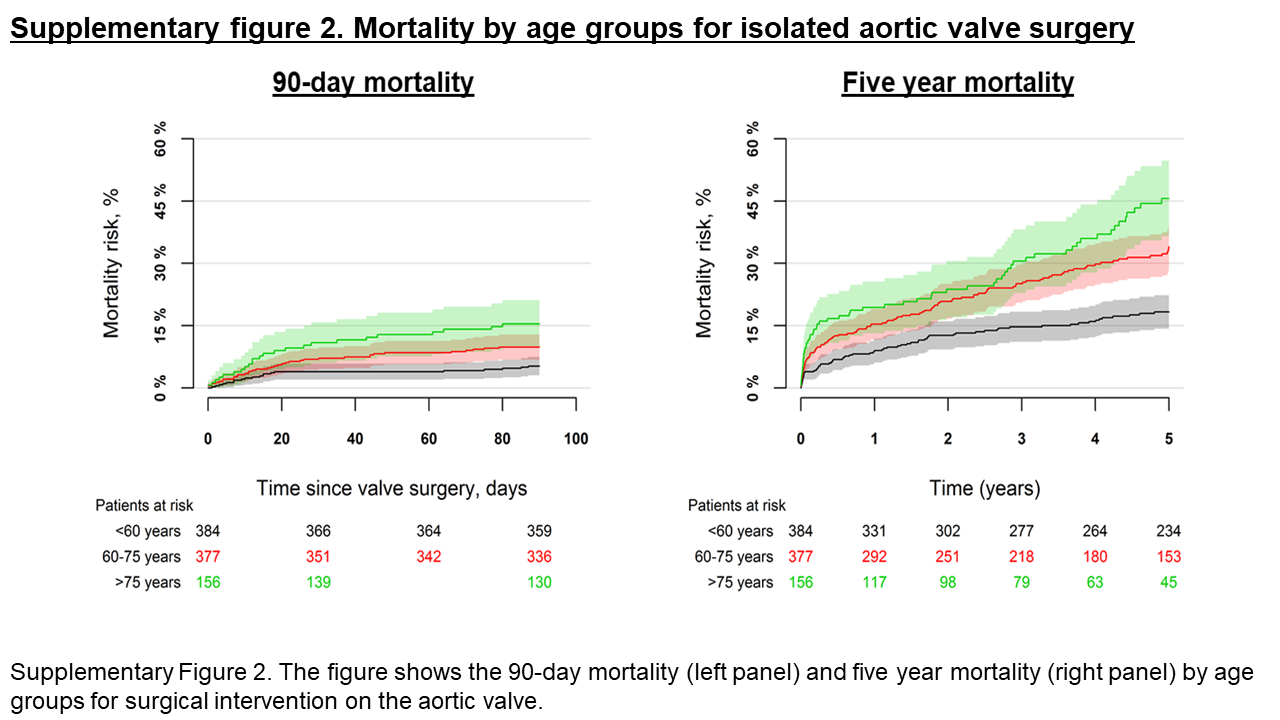

Supplement: Supplementary file 2 — Additional file 2: Supplementary Figure 2. Mortality by age groups for isolated aortic valve surgery. The figure shows the 90-day mortality (left panel) and 5 year mortality (right panel) by age groups for surgical intervention on the aortic valve. [file 12879_2020_5422_MOESM2_ESM.tif]

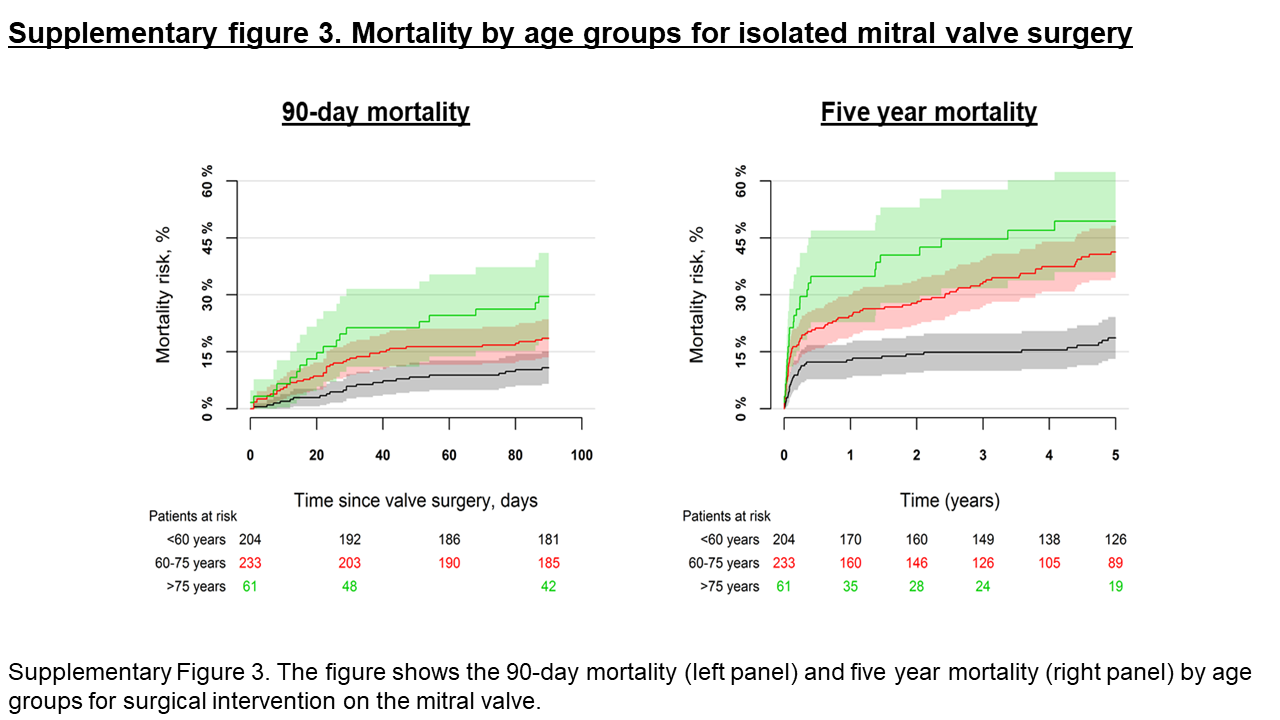

Supplement: Supplementary file 3 — Additional file 3: Supplementary Figure 3. Mortality by age groups for isolated mitral valve surgery. The figure shows the 90-day mortality (left panel) and 5 year mortality (right panel) by age groups for surgical intervention on the mitral valve. [file 12879_2020_5422_MOESM3_ESM.tif]

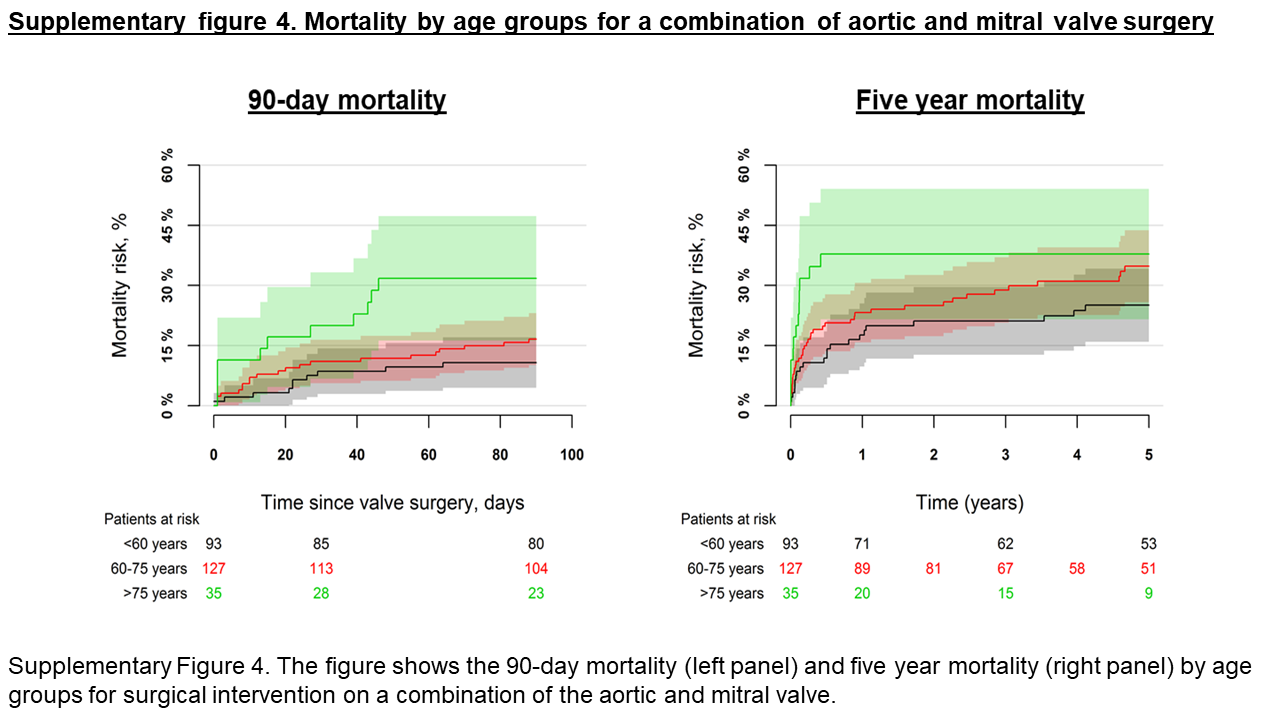

Supplement: Supplementary file 4 — Additional file 4: Supplementary Figure 4. Mortality by age groups for a combination of aortic and mitral valve surgery. The figure shows the 90-day mortality (left panel) and 5 year mortality (right panel) by age groups for surgical intervention on a combination of the aortic and mitral valve. [file 12879_2020_5422_MOESM4_ESM.tif]
