# Supplementary material for: Differences in mortality in patients undergoing surgery for infective endocarditis according to age and valvular surgery
Source: BMC Infect Dis. 2020 Sep 25;20:705. doi: 10.1186/s12879-020-05422-8 (PMC7519559; doi:10.1186/s12879-020-05422-8)
Supplement: Supplementary file 5 — Additional file 5. Supplementary material, Results continued. [file 12879_2020_5422_MOESM5_ESM.docx]

**Supplementary material, Results continued**

*Isolated aortic valve surgery*
Of the 917 patients undergoing isolated aortic valve surgery 99.2% underwent an aortic valve replacement. Including a total of 12 redo procedures conducted during IE admission, 321 patients (35.0%) underwent a mechanical aortic valve replacement, 494 patients (53.9%) underwent a bioprosthetic aortic valve replacement, and 114 patients (12.4%) underwent other aortic valve replacement. The in-hospital mortality for patients undergoing isolated aortic valve surgery was 4.4%, 9.6%, and 13.5% for patients <60 years, 60-75 years, and ≥75 years of age, respectively (p=0.0009). In adjusted analysis, we found an OR of 1.87 (95% CI: 0.97-3.61) and 2.88 (95% CI: 1.35-6.14) for patients 60-75 years and ≥75 years, respectively compared with patients <60 years. The 90-day mortality was 5.4%, 9.8%, and 15.6% (p=0.0005), respectively while the five year mortality was 19.0%, 34.1%, and 45.3%, respectively (p<0.0001), Supplementary Figure 2. In adjusted analysis, patients 60-75 years and ≥75 years were associated with a consistent higher mortality during follow-up of up to five years, HR=1.80 (95% CI: 1.30-2.50) and HR=2.42 (95% CI: 1.64-3.57) as compared with patients <60 years.

*Isolated mitral valve surgery*
Of the 498 patients undergoing isolated mitral valve surgery 74.1% underwent a mitral valve replacement and 25.9% underwent mitral valve surgery without replacement. Including a total of six valve replacement redo procedures conducted during IE admission, 176 patients (35.3%) underwent a mechanical mitral valve replacement and 199 patients (40.0%) underwent a bioprosthetic mitral valve replacement. The in-hospital mortality for patients undergoing isolated mitral valve surgery was 8.3%, 16.7%, and 24.6% for patients <60 years, 60-75 years, and ≥75 years of age, respectively (p=0.002). In adjusted analysis, we found an OR of 2.29 (95% CI: 1.21-4.32) and 3.50 (95% CI: 1.55-7.88) for patients 60-75 years and ≥75 years, respectively compared with patients <60 years. The 90-day mortality was 10.8%, 18.5%, and 29.6% (p=0.001), respectively and the five year mortality was 18.6%, 41.3%, and 49.4%, respectively (p<0.0001), Supplementary Figure 3. In adjusted analysis, patients 60-75 years and ≥75 years were associated with a consistent higher mortality during follow-up of up to five years, HR=2.54 (95% CI: 1.70-3.80) and HR=3.53 (95% CI: 2.11-5.89) as compared with patients <60 years.

*Combination of aortic and mitral valve surgery*
Of the 255 patients undergoing a combination of aortic and mitral valve surgery 96.1% and 70.6% had an aortic valve replacement and a mitral valve replacement, respectively. In total, eight valve replacement redo procedures were conducted during IE admission. We found that 80 patients (31.4%) underwent a mechanical aortic valve replacement, 145 (56.9%) underwent a bioprosthetic aortic valve replacement, and 27 patients (10.6%) underwent other aortic valve replacement. Further, 66 patients (25.9%) underwent a mechanical mitral valve replacement and 115 patients (45.1%) underwent a bioprosthetic mitral valve replacement. In-hospital mortality was 11.8%, 18.1%, 34.3% for patients <60 years, 60-75 years, and ≥75 years of age, respectively (p=0.01). In adjusted analysis, we found an OR of 1.40 (95% CI: 0.58-3.38) and 3.27 (95% CI: 1.10-9.71) for patients 60-75 and ≥75 years, respectively compared with patients <60 years. The 90-day mortality was 9.0%, 16.0%, and 36.3% (p=0.01), respectively while the five year mortality was 25.1%, 34.8%, and 37.8%, respectively (p=0.11), Supplementary Figure 3.

In adjusted analysis, we found no significant difference in the associated risk of five year mortality by age groups, HR=1.08 (95% CI: 0.60-1.95) and HR=1.53 (95% CI: 0.71-3.29) for patients 60-75 years and ≥75 years compared with patients <60 years.

We identified that 13.7% of patients undergoing a combination of mitral and aortic valve surgery had a length of hospital stay >90-day. For isolated aortic valve surgery and isolated mitral valve surgery this was 7.0% and 11.0%, respectively.
