# Supplementary material for: Differences in mortality in patients undergoing surgery for infective endocarditis according to age and valvular surgery
Source: BMC Infect Dis. 2020 Sep 25;20:705. doi: 10.1186/s12879-020-05422-8 (PMC7519559; doi:10.1186/s12879-020-05422-8)
Supplement: Supplementary file 6 — Additional file 6: Supplementary Table 1. Codes. [file 12879_2020_5422_MOESM6_ESM.docx]

| **Supplementary Table 1: Codes** | | |
| --- | --- | --- |
| **Category** |  | **Codes** |
|  |  |  |
| Study population |  |  |
| Infective endocarditis |  | ICD-10: DI33, DI38, DI398; ICD8: 421 |
| Aortic valve surgery |  | KFM, KFCA60, KFCA70, KFK. Mechanical aortic valve prosthesis: KFMD00 and KFCA60.  Bioprosthetic aortic valve prosthesis: KFMD10, KFMD11, KFMD14, KFCA70. |
|  |  | Other aortic valve replacement: KFMD20, KFMD30, KFMD33, KFMD40, KFMD96. |
| Mitral valve surgery |  | KFK  Mechanical mitral valve replacement: KFKD00.  Bioprosthetic mitral valve replacement: KFKD10. |
| Right-sided valve surgery |  | KFG and KFJ |
|  |  |  |
| Comorbidity |  |  |
| Acute myocardial infarction |  | ICD-10: I21-22; ICD-8: 410. |
| Heart failure |  | ICD-10: DI42, DI50, DI110, DJ819; ICD-8: 4270, 4271. |
| Atrial flutter/fibrillation |  | ICD-10: DI48; ICD-8: 4274. |
| Mitral valve disease |  | ICD-10: DI34; ICD-8: 394, 396 |
| Aortic valve disease |  | ICD-10: DI35; ICD-8: 395, 396 |
| CIED |  | BFCA0 and BFCB0 |
| Prosthetic heart valve |  | KFKD, KFMD, KFGE, KFJF |
| Renal disease |  | ICD10: DN03-04, DN17-19, DR34, DI12-13; ICD-8: 582-586, 588. |
| Renal dialysis |  | ICD-10: Z992. BJFD2 |
| Peripheral vascular disease |  | ICD-10: I70-73, R02, I771; ICD-8: 443. |
| Cerebrovascular disease |  | ICD-10: I60-69; ICD8: 430-438. |
| Cancer |  | ICD-10: DC00-DC97; ICD-8: 140-209 |
| Chronic obstructive lung disease |  | ICD10: DJ42, DJ44; ICD-8: 490-492 |
| Liver disease |  | ICD-10: K70-77, B150, B160, B190; ICD-8: 571, 572, 456. |
|  |  |  |
| Pharmacotherapy |  |  |
| Diuretics |  | ATC code: C03 |
| Beta blockade |  | ATC code: C07 |
| RAS-inhibition |  | ATC code: C09 |
| Lipid lowering medication |  | ATC code: C10 |
| Corticosteroid medication |  | ATC code: H02 |
| Aspirin |  | ATC codes: B01AC06, N02BA01 |
| Anticoagulants |  | ATC code: B01AA, B01AE07, B01AF01, B01AF02 |
| Antibiotics |  | ATC code: J01 |
| Diabetes (glucose lowering medication) |  | ATC code: A10 |
| ICD: international classification of diseases, ATC: Anatomical Therapeutical Classification System, RAS: renin angiotensin system, CIED: cardiac implantable electronic device. | | |
