# Supplementary material for: Differences in mortality in patients undergoing surgery for infective endocarditis according to age and valvular surgery
Source: BMC Infect Dis. 2020 Sep 25;20:705. doi: 10.1186/s12879-020-05422-8 (PMC7519559; doi:10.1186/s12879-020-05422-8)
Supplement: Supplementary file 7 — Additional file 7: Supplementary Table 2. Baseline characteristics for patients undergoing left-sided valve surgery for IE. [file 12879_2020_5422_MOESM7_ESM.docx]

| **Supplementary Table 2. Baseline characteristics for patients undergoing left-sided valve surgery for IE** | | | | | |
| --- | --- | --- | --- | --- | --- |
|  | **All** | **Aortic valve** | **Mitral valve** | **Aortic and mitral valve** |  |
| Number | 1,670 | 917 | 498 | 255 |  |
| Age, median (IQR) | 63.2 [53.3, 71.7] | 62.9 [53.2, 72.3] | 63.1 [53.2, 70.7] | 64.7 [55.2, 71.6] | 0.38 |
| Female, N (%) | 436 (26.1) | 202 (22.0) | 170 (34.1) | 64 (25.1) | <0.001 |
|  |  |  |  |  |  |
| Medical history prior to IE admission, N (%) | | | | | |
| AMI | 95 (5.7) | 60 (6.5) | 25 (5.0) | 10 (3.9) | 0.21 |
| Heart failure | 202 (12.1) | 120 (13.1) | 60 (12.0) | 22 (8.6) | 0.15 |
| Afib | 226 (13.5) | 125 (13.6) | 60 (12.0) | 41 (16.1) | 0.31 |
| Mitral valve disease | 157 (9.4) | 20 (2.2) | 106 (21.3) | 31 (12.2) | <0.001 |
| Aortic valve disease | 431 (25.8) | 339 (37.0) | 30 (6.0) | 62 (24.3) | <0.001 |
| CIED | 55 (3.3) | 36 (3.9) | 14 (2.8) | 5 (2.0) | 0.23 |
| Prosthetic heart valve | 206 (12.3) | 149 (16.2) | 29 (5.8) | 28 (11.0) | <0.001 |
| Renal disease | 118 (7.1) | 52 (5.7) | 46 (9.2) | 20 (7.8) | 0.04 |
| Dialysis | 25 (1.5) | 14 (1.5) | 9 (1.8) | 2 (0.8) | 0.55 |
| Peripheral vascular disease | 146 (8.7) | 96 (10.5) | 32 (6.4) | 18 (7.1) | 0.02 |
| Cerebrovascular disease | 154 (9.2) | 88 (9.6) | 45 (9.0) | 21 (8.2) | 0.79 |
| Cancer | 185 (11.1) | 111 (12.1) | 47 (9.4) | 27 (10.6) | 0.30 |
| COPD | 120 (7.2) | 70 (7.6) | 33 (6.6) | 17 (6.7) | 0.74 |
| Liver disease | 54 (3.2) | 28 (3.1) | 17 (3.4) | 9 (3.5) | 0.90 |
| Diabetes | 177 (10.6) | 91 (9.9) | 56 (11.2) | 30 (11.8) | 0.60 |
|  |  |  |  |  |  |
| Medication six months prior to IE admission, N (%) | | | | | |
| Diuretics | 510 (30.5) | 303 (33.0) | 130 (26.1) | 77 (30.2) | 0.03 |
| Beta blockade | 386 (23.1) | 221 (24.1) | 104 (20.9) | 61 (23.9) | 0.37 |
| RAS inhibition | 522 (31.3) | 302 (32.9) | 132 (26.5) | 88 (34.5) | 0.02 |
| Lipid lowering medication | 412 (24.7) | 239 (26.1) | 109 (21.9) | 64 (25.1) | 0.22 |
| Corticosteroids | 130 (7.8) | 81 (8.8) | 29 (5.8) | 20 (7.8) | 0.13 |
| Aspirin | 352 (21.1) | 226 (24.6) | 78 (15.7) | 48 (18.8) | <0.001 |
| Anticoagulants | 281 (16.8) | 166 (18.1) | 68 (13.7) | 47 (18.4) | 0.08 |
| Antibiotics | 844 (50.5) | 472 (51.5) | 239 (48.0) | 133 (52.2) | 0.39 |
| Right-sided valve intervention excluded. IE: infective endocarditis, IQR: interquartile range, 25^th^ and 75^th^ percentile, AMI: acute myocardial infarction, Afib: atril fibrillation/flutter, CIED: cardiac implantable electronic device, COPD: chronic obstructive pulmonary disease, RAS: renin-angiotensin system | | | | | |
