# Supplementary material for: Differences in mortality in patients undergoing surgery for infective endocarditis according to age and valvular surgery
Source: BMC Infect Dis. 2020 Sep 25;20:705. doi: 10.1186/s12879-020-05422-8 (PMC7519559; doi:10.1186/s12879-020-05422-8)
Supplement: Supplementary file 8 — Additional file 8: Supplementary Table 3. The table shows the mortality by sub groups. [file 12879_2020_5422_MOESM8_ESM.docx]

| **Supplementary Table 3. The table shows the mortality by sub groups** | | | |
| --- | --- | --- | --- |
|  | In-hospital mortality, % | 90 day mortality, % | Five years mortality, % |
| Total study population, N=1,767 | 11.6 | 12.5 | 30.8 |
| <60 years, N=735 | 6.4 | 7.5 | 19.7 |
| 60-75 years, N=766 | 13.6 | 13.9 | 37.0 |
| ≥75 years, N=266 | 20.3 | 22.3 | 46.2 |
|  |  |  |  |
| Isolated aortic surgery, N=917 | 8.1 | 8.9 | 28.9 |
| <60 years, N=384 | 4.4 | 5.4 | 19.0 |
| 60-75 years, N=377 | 9.6 | 9.8 | 34.1 |
| ≥75 years, N=156 | 13.5 | 15.6 | 45.3 |
|  |  |  |  |
| Isolated mitral valve surgery, N=498 | 14.3 | 16.7 | 32.7 |
| <60 years, N=204 | 8.3 | 10.8 | 18.6 |
| 60-75 years, N=233 | 16.7 | 18.5 | 41.3 |
| ≥75 years, N=61 | 24.6 | 29.6 | 49.4 |
|  |  |  |  |
| Mitral+aortic valve surgery, N=255 | 18.3 | 16.5* | 31.6 |
| <60 years, N=93 | 11.8 | 9.0* | 25.1 |
| 60-75 years, N=127 | 18.1 | 16.0* | 34.8 |
| ≥75 years, N=35 | 34.3 | 36.3 | 37.8 |
| *The median duration of admission were more than 90 days  Mortality at 90 days and five years were estimated using Kaplan Meier estimates. | | | |
